# Rejuvenating the gut: young plasma therapy improves cell proliferation, IGF-I and IGF-IR expression, and immune defense in aged male rats jejunum

**DOI:** 10.1007/s10522-025-10204-3

**Published:** 2025-02-19

**Authors:** Ender Deniz Asmaz, Murat Tan, Aysun Inan Genç, Hikmet Taner Teker, Taha Ceylani

**Affiliations:** 1 Faculty of Medicine, Department of Histology and Embryology, Ankara Medipol University, Ankara, Turkey; 2 Department of Electrical&Computer Engineering, Boston University, Biomedical Engineering Graduate Medical Sciences, Boston, MA 02215 USA; 3 Department of General Surgery, Istanbul Demiroglu Bilim University, Istanbul, Turkey; 4 Faculty of Science, Department of Biology, Kastamonu University, Kastamonu, Turkey; 5 Faculty of Medicine, Department of Medical Biology and Genetics, Ankara Medipol University, Ankara, Turkey; 6 Department of Food Processing, Muş Alparslan University, Muş, Turkey; 7 Department of Molecular Biology and Genetics, Muş Alparslan University, Muş, Turkey

**Keywords:** Young plasma, Jejunum, Proliferation, IGF-I, IGF-IR, IgA

## Abstract

It is well known that aging affects many systems in the body. The digestive system is one of the systems most affected by aging. In our study, we examined the effects of young plasma treatment on cell proliferation, growth factors, immune defense and histological parameters in the jejunum of aged male rats. For this purpose, aged male Sprague Dawley rats (24 months, n = 7) were treated with pooled plasma (0.5 ml/day, intravenously for 30 days) collected from young (5 weeks, n = 51) rats. Aged rats that received young plasma treatment were grouped as the experimental group, while aged rats formed the control group. At the end of the experiment, the jejunums of the groups were collected and histological parameters such as villus height, crypt depth, total mucosal thickness and surface absorption areas were measured and compared. In addition, cell proliferation index and proliferation intensity in the crypt glands of the jejunum were evaluated with proliferating cell nuclear antigen and expressions of growth factors such as insulin-like growth factor I (IGF-I) and its receptor (IGF-IR) expression and effects of immunoglobulin A (IgA), which plays a role in the defense of the digestive system against microorganisms, were examined. In the experimental group, an increase in histological parameters, IGF-R and IGF-IR expression, proliferation density, proliferation index and IgA expression density and IgA cell count were observed compared to the control group. These results suggest that young plasma treatment has a positive effect on the digestive system and may be a potential therapeutic for tissue regeneration.

## Introduction

Aging is characterized by a gradual slowing down of normal physiological functions throughout life. With age, each organ system in the body gradually loses its resistance and the individual becomes more susceptible to many diseases (Wang et al. 2022). Numerous studies have revealed how aging occurs and how it is regulated by complex cellular mechanisms. Many factors have been reported to affect the aging process and longevity (Sun et al. 2022; Cai et al. 2022). Young blood plasma therapy is becoming an attractive treatment approach at this point. Its potential therapeutic benefits and claims to reduce age-related damage are increasing day by day. Recent studies suggests that systemic factors present in young blood contribute to tissue regeneration, prompting investigations into its therapeutic applications across multiple organ systems (Loffredo et al. 2013; Allahverdi 2024). Studies have demonstrated that young plasma can modulate progenitor cell activity (Conboy et al. 2005), enhance neurogenesis (Villeda et al. 2011), and increase antioxidant enzyme activity in aged organisms (Tripathi et al. 2021).

The digestive system, particularly the intestines, has a critical role in overall health, facilitating nutrient and water absorption, energy production, and waste elimination (Guler et al. 2022). As we age, the gastrointestinal system, which is associated with many organ systems, undergoes various morphological and functional changes (Merchant et al. 2014), which can disrupt normal homeostatic mechanisms. Studies on the digestive system in particular have focused primarily on the histomorphometric parameters of the digestive system organs (Asmaz and Seyidoglu 2022; Ruttanavut and Yamauchi 2010). Studies have emphasized the importance of proliferation in crypt glands in intestinal regeneration, and have frequently evaluated proliferative markers such as Proliferating Cell Nuclear Antigen (PCNA) and Ki-67 (Asmaz et al. 2025a; Guler et al. 2022; Garcia et al. 2007).

On the other hand, a significant decrease in the secretion of growth factors is observed with aging (Kuemmerle 2012; Pollak et al. 2004; Firth and Baxter 2002). Insulin-like growth factor (IGFs), one of the growth factors effective especially in the digestive system, is associated with decreased intestinal regeneration and adaptive responses (Kuemmerle 2012; Pollak et al. 2004). Insulin-like growth factor (IGF-I), the main mediator of trophic effects of growth hormone, is mitogenic for intestinal epithelial and smooth muscle cells and hepatic stellate cells and promotes adaptive mucosal proliferation both physiologically and pathophysiologically (Dahly et al. 2003; Mantell et al. 1995). In particular, downregulation of IGF-I expression with age highlights its potential role in gastrointestinal aging and dysfunction and draws attention to its therapeutic effect on aging.

The role of immune system on the digestive system is the most important part of the human immune system. The weakening of the immune system with age causes a decrease in resistance to pathogens. This leads to an increase in morbidity and mortality from infections in old age (Beharka et al. 2001). Immune system-associated IgA regulates the composition of the intestinal microbiota and plays an important function in intestinal homeostasis by preventing microbial translocation (Corthésy 2013). However, studies have provided conflicting reports of a decrease or no change in cellular immunity with age (Gupta et al. 2023). Therefore, there is a need to investigate the relationship between a novel anti-aging therapy and immunity.

In our previous studies, we obtained results showing that middle-aged female rat plasma induces duodenal cell proliferation in aged tissues (Asmaz et al. 2025b). In the present study, we took these results one step further and aimed to determine the effects of plasma collected from young male rats on histomorphological parameters, cell proliferation index and cell proliferation density, IGF-I and IGF-IR expressions in the jejunum, and the relationship between anti-aging treatment and immunity (immunoglobulin A (IgA)) in terms of both IgA cell count and IgA expression density. This study suggests that young plasma therapy will increase cellular proliferation, restore IGF-I/IGF-IR signaling, and improve intestinal immunity through increased IgA expression, thus reducing age-related degeneration in the gastrointestinal tract.

## Materials and methods

### Animal studies

The study was conducted with the approval of the Ethics Committee of the Saki Yenilli Experimental Animal Production and Application Laboratory (Approval number: 2021/03) and was performed with the National Institutes of Health Guide for the Care and Use of Laboratory Animals.

In the study, aged male Sprague Dawley rats (24 months, control group; n = 7) were treated with pooled plasma (0.5 ml/day, intravenously for 30 days) collected from young (5 weeks, n = 51) rats. Aged rats that received young plasma treatment were labeled as the experimental group (24 months, n = 7), while aged rats formed the control group (24 months, n = 7). The transferred blood plasma was determined according to 1/10 of the animal's blood plasma amount (Ceylani and Teker 2022). Rats in each group were kept in separate cages and were kept in transparent Plexiglas cages (5 rats per cage) with free access to food and water under a 12-h light/dark cycle at a constant 21 °C temperature. No animals were lost during the experiment. All animals were sacrificed under ether anesthesia. Their jejunum was collected and placed in fixation solution for histological study.

### Plasma collection

Pooled rat plasma was collected by terminal cardiac puncture during euthanasia. Plasma was prepared from blood collected with EDTA, followed by centrifugation at 1000 *g*. For plasma denaturation, plasma was heated for 2–3 min at 95 °C, followed by a short spin at 1000 g. All plasma aliquots were stored at − 80 °C until use. Before administration, plasma was dialyzed using 3.5-kDa D-tube dialyzers (EMD Millipore) in PBS to remove EDTA (Ceylani et al. 2023a).

### Histological analyzes of the jejunum

The jejunums of sacrificed animals were taken and placed in 10% buffered neutral formalin solution, fixed for 24 h. The jejunums that were examined manually the next day and found to be fixed were placed in tap water to remove the fixative. They were then passed through increasing alcohol series and blocked immediately after paraffin impregnation. Sections obtained from paraffin blocks were stained with Crossmonn triple stain determine morphological changes in tissue samples by light microscopy (Carl Zeiss-GmbH ZEN 3.5 Software) (Crossmon 1937). Morphometric parameters were measured as villus height (µm), crypt depth (µm), total mucosal thickness (µm) and surface absorption area (mm^2^). Villus absorption surface area was calculated using the following formula: Villus absorption surface area = 2 π × (average villus width/2) × villus height. Immunohistochemical method was used to determine cell proliferation (Yesilbag et al. 2022).

### Immunohistochemical analysis

Sections were stained with the indirect streptavidin–biotin-peroxidase complex method and evaluated under a light microscope. After the sections were deparaffinized, they were passed through an alcohol series. For the antigen retrieval stage, 2.1 g of citric acid was dissolved in 1 L of distilled water and the pH was adjusted to 6 and the tissues were treated in a 750 W microwave (Arçelik MD 524) for 3 × 5 min. In order to block endogenous peroxidase activity, 3% hydrogen peroxide solution was used and in order to prevent nonspecific protein binding, secondary blocking serum was used. Primary antibodies were applied and incubated overnight at + 4 °C (PCNA: sc-7907; Santa Cruz, dilution:1/200—IGF-I: G-17 sc-1422; Santa Cruz, dilution:1/100—IGF-IR:C-20 sc: 713; Santa Cruz, dilution:1/100—Goat-Anti-Rat IgA: ab97185; Abcam, dilution:1/200). Negative controls were incubated with the antibody diluent without using the primary antibody (control groups jejunum). The tissues were incubated with secondary antibody (ImmPRESS reagent Vector Laboratories, for IGF-I: MP7405, for IGF-IR, PCNA and IgA: MP7401) for 30 min the next day. After imaging with 3,3'-diaminobenzidine (DAB-Zymed Laboratories, USA) chromogen, they were then counterstained with Harris Hematoxylin and covered with entellan.

### Evaluation

Proliferative index (PI) was obtained by calculating the ratio of the number of PCNA positive crypt cells to the total number of crypt cells. It was defined as the average of the proliferating cell numbers in 15 randomly selected crypts from the sections (Asmaz and Seyidoglu 2022).

To calculate the mean values of IgA + cells, IgA + cells were counted for 15 randomly selected areas from five sections per group and the mean IgA + cell numbers per view (over areas of 0.01 mm^2^) were calculated for each group (Yang et al. 2021).

In addition, the localization and intensity of PCNA (in the crypt), IGF-I and IGF-IR (in the villus and crypt) expressions were also evaluated by two independent observers. In the evaluation made according to the scoring system, 0 means no immunoreactivity; (1) means weak immunoreactivity; (2) means moderate immunoreactivity; (3) means strong immunoreactivity (Asmaz et al. 2022; Zık et al. 2019).

### Statistical analysis

Since parametric test assumptions were not provided as descriptive statistics for numerical variables in the study, the results were given as median (minimum–maximum) values using the Mann Witney U test. In the analyses, p < 0.05 was considered statistically significant. IBM SPSS v29 program was used for the analyses of the study.

## Results

At the end of the experiment, both morphometric data and immunohistochemical data of the jejunum were evaluated.

### Morphometric evaluation

At the end of the experiment, the jejunums of the experimental group and control group were collected and evaluated according to histomorphological parameters. The histomorphological parameters evaluated were; villus height, crypt depth, total mucosal thickness, surface absorption area.

**Fig. 1:**
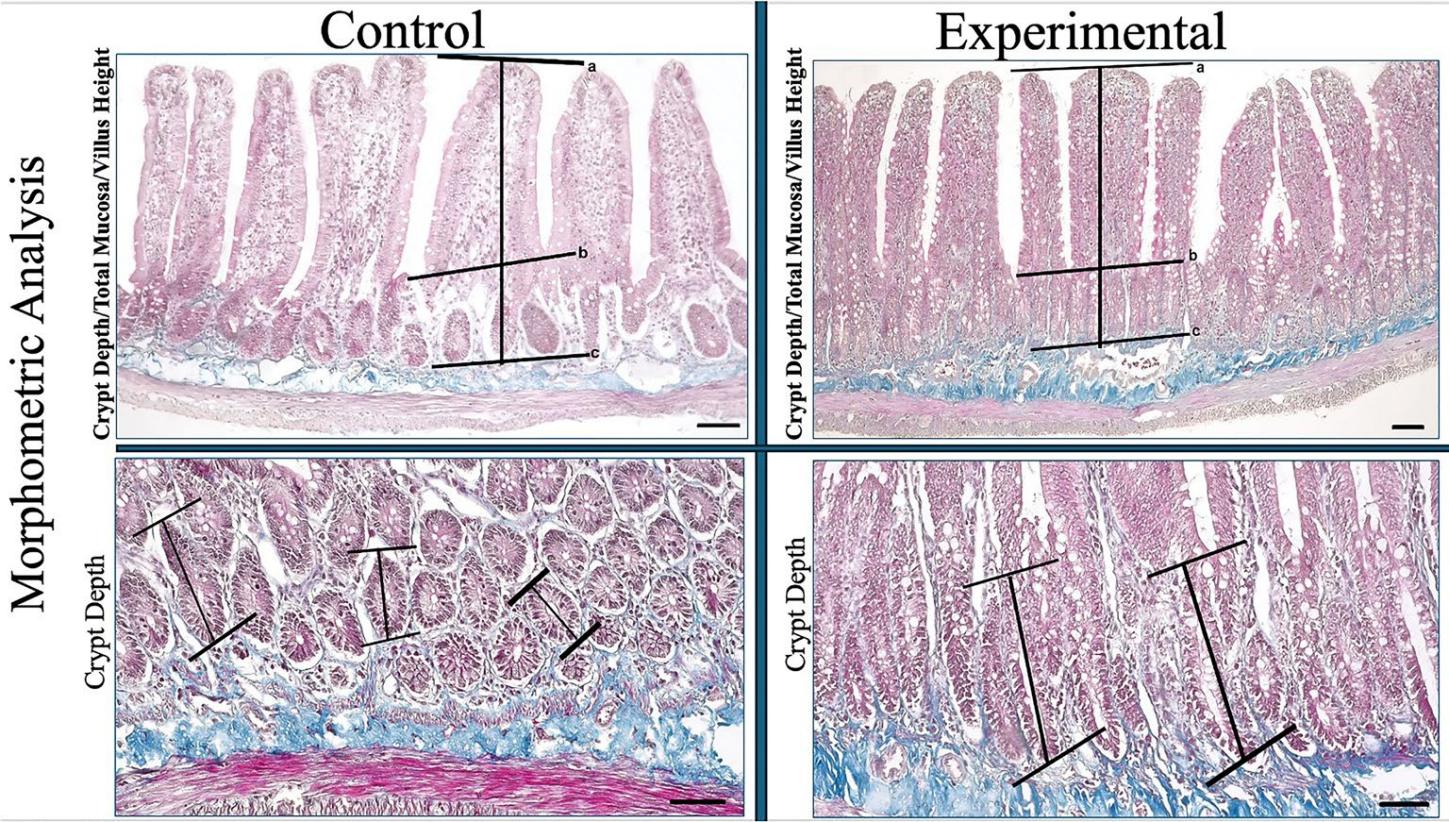
Morphometric analysis of jejunum in experimental and control groups. **a–b** Villus height, **b–c** crypt depth, **a–c** total mucosal thickness (Bar: 50 µm)

The experimental group showed an increase in histological parameters, (Villus Height; control: 960.2 µm—experimental: 1197.6 µm p < 0.001, Crypt Depth; control: 234.2 µm—experimental: 351.4 µm p < 0.001, Total Mucosa; control: 1290.6 µm—experimental: 1509.7 µm p < 0.001, Surface Area; control: 1.10 µm—experimental: 1.47 µm p < 0.001) compared to the control group (Fig. 1) (Table 1).

**Table 1:** Morphometric analysis of the villus height, crypt depth, total mucosal thickness and villus surface absorption area of the control and experimental groups jejunum

Groups	N	Villus height	Crypt depth	Total mucosal thickness	Surface absorption area
Control	7	960.2(980.2–920.8)^a^	234.2(220.4–255.3)^a^	1290.6(1210,4–1322.6)^a^	1.10(1.0–1.12)^a^
Experimental	7	1197.6(1182.2–1218.4)^b^	351.4(330.6–362.4)^b^	1509.7(1430.8–1528.4)^b^	1.47(1.38–1.52)^b^
P value		< *0.001*	< *0.001*	< *0.001*	< *0.001*

Different letters in the same column show statistical significance (^a,b^)

### Immunohistochemical evaluation

At the end of the experiment, PCNA proliferation intensity and proliferation index (PI), IgA expression intensity and IgA cell count, IGF-I, IGF-IR expression intensity were evaluated in the jejunum of the control and experimental groups.

**Fig. 2:**
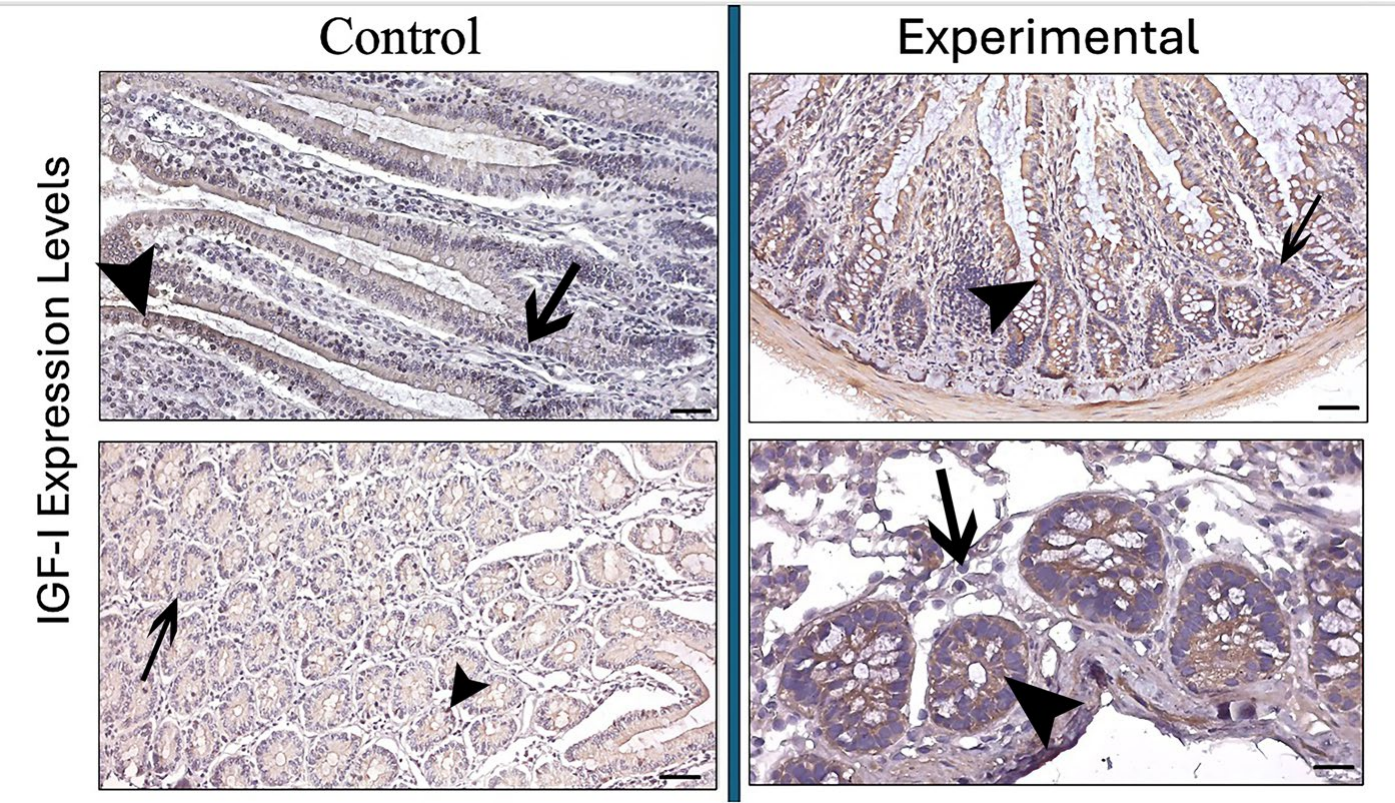
IGF-I expression in the jejunum of control and experimental groups. Arrowhead: IGF-I positive immunoreaction, arrow: IGF-I negative immunoreaction. (Bar: 50 µm)

IGF-I expression intensity was evaluated in both jejunal villi and crypt glands. While IGF-I expression was determined as weak in aged rat villi, IGF-I expression showed a moderate level of expression intensity in the villi of rats in the experimental group treated with young plasma (control: 1.68 µm—experimental: 2.20 µm p < 0.001). When the crypt glands were examined, a weak IGF-I immunoreaction was observed in the control group, while this expression intensity was determined as moderate in the treatment group (control: 1.50 µm—experimental: 2.32 µm p < 0.001) (Fig. 2) (Table 2).

**Table 2:** IGF-I and IGF-IR expression in control and experimental groups jejunum

Groups	N	IGF-I Expression (villus)	IGF-I Expression (crypt)	IGF-IR Expression (villus)	IGF-IR Expression (crypt)
Control	7	1.68(1.55–1.90)^a^	1.50(1.42–1.64)^a^	1.90(1.78–2.00)^a^	1.80(1.68–1.90)^a^
Experimental	7	2.20(2.12–2.30)^b^	2.32(2.20–2.44)^b^	2.42(2.34–2.50)^b^	2.30(2.22–2.46)^b^
P value		< 0.001	< 0.001	< 0.001	< 0.001

Different letters in the same column show statistical significance (^a,b^)

**Fig. 3:**
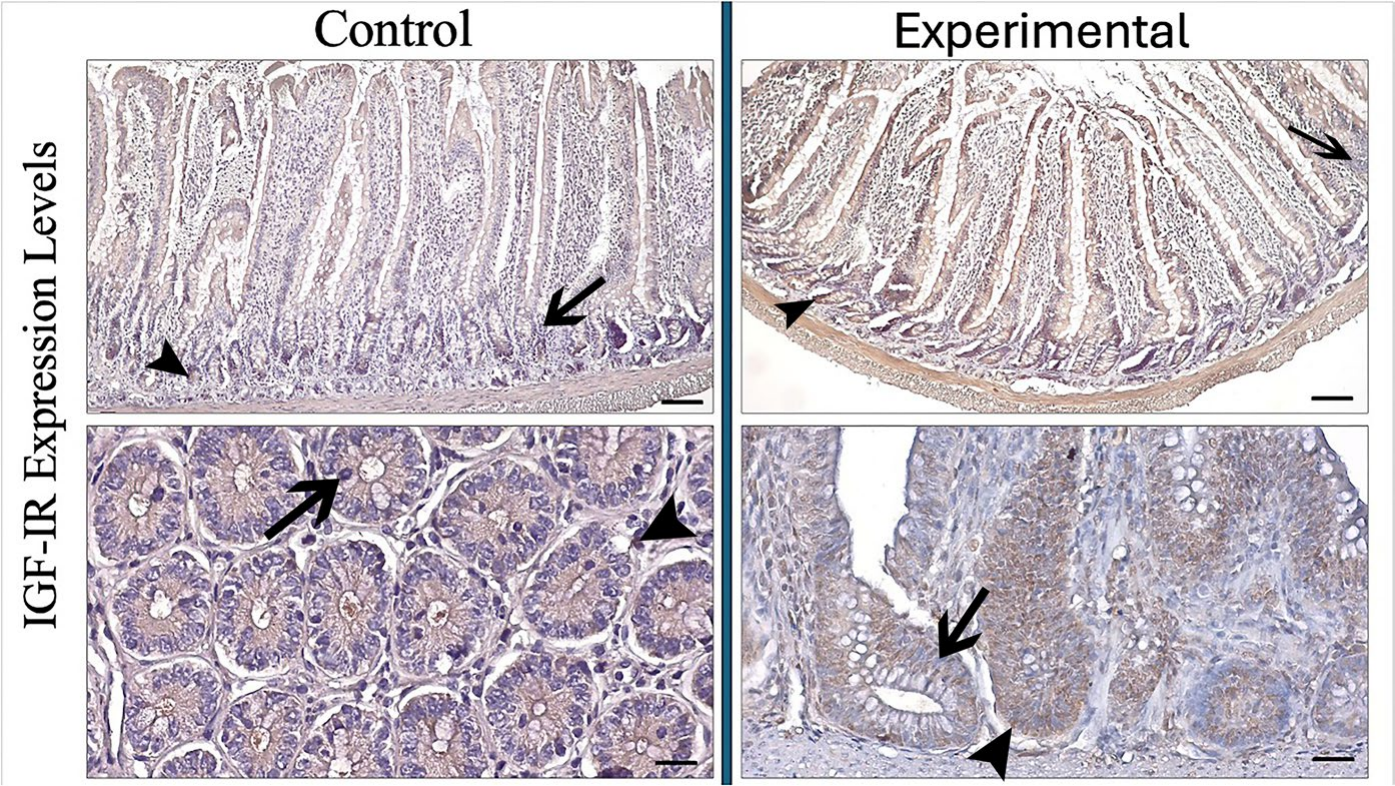
IGF-IR expression in the jejunum of control and experimental groups. Arrowhead: IGF-I positive immunoreaction, arrow: IGF-IR negative immunoreaction. (Bar: 50 µm)

IGF-IR expression evaluated in both jejunal villi and crypt glands was consistent with the increase in IGF-I expression. IGF-IR expression in the villi of jejunal tissue of aged rat showed low-moderate expression, while moderate-high IGF-IR expression was determined in the villi of experimental group (control: 1.90 µm—experimental: 2.42 µm p < 0.001). In crypt glands, IGF-IR expression showed a low immunoreaction in control group, while moderate immunoreaction in experimental group (control: 1.80 µm—experimental: 2.30 µm p < 0.001) (Fig. 3) (Table 2).

**Fig. 4:**
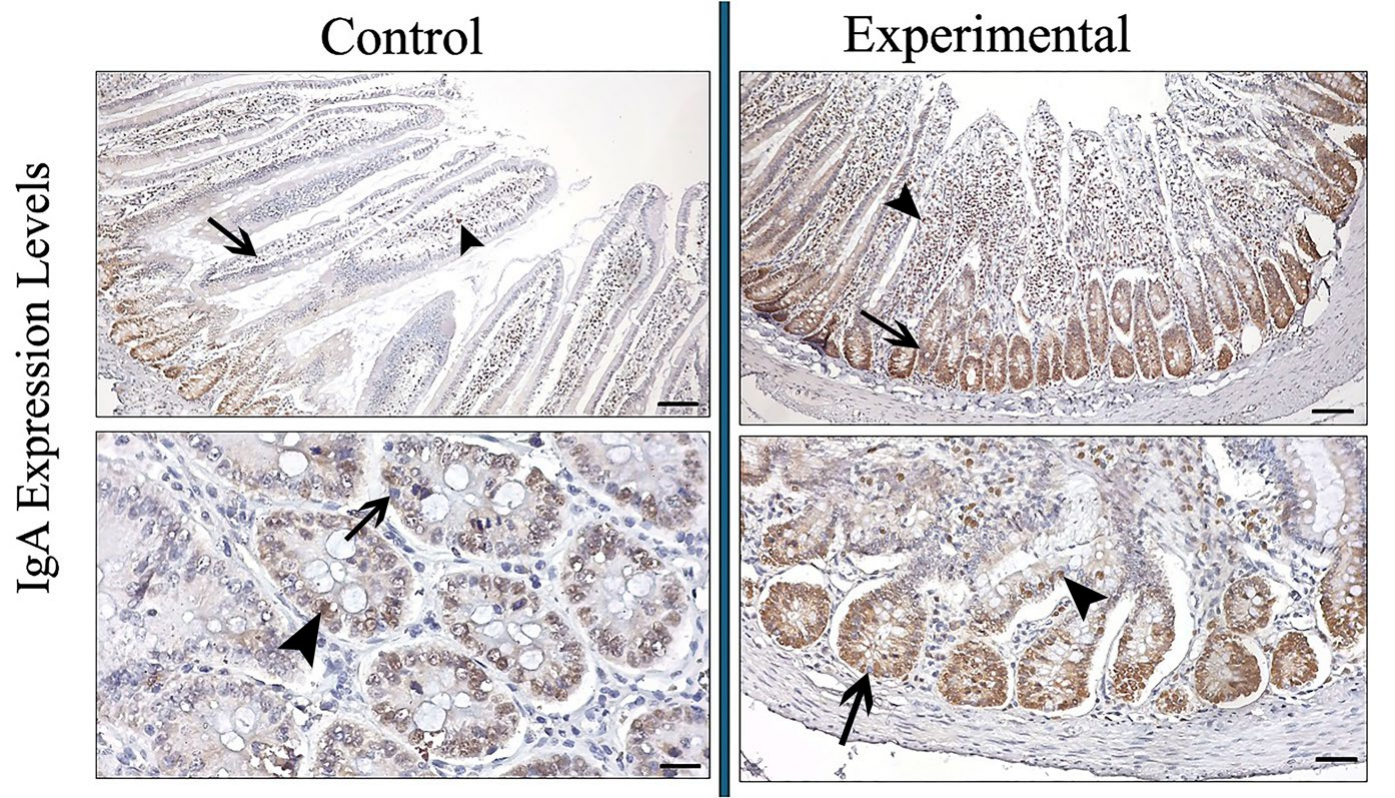
IgA expression in the jejunum of control and experimental groups. Arrowhead: IgA positive cells, arrow: IgA negative cells. (Bar: 50 µm)

IgA was expressed in jejunal villi and crypt glands. While IgA expression was determined at a moderate level in the experimental group, statistically low IgA expression was seen in the control group (control: 1.62 µm—experimental: 2.50 µm p < 0.001) (Fig. 4) (Table 3).

**Table 3:** PCNA expression intensity, proliferation index (PI), IgA expression intensity and IgA cell count in control and experimental groups jejunum

Groups	N	PCNA expression	Proliferation index (PI)	IgA expression	IgA cell count
Control	7	1.65(1.50–1.80)^a^	34.22(28.12–40.20)^a^	1.62(1.50–1.75)^b^	84.11(72.12–96.10)^a^
Experimental	7	2.75(2.65–2.84)^b^	85.12(68.12–94.10)^b^	2.50(2.38–2.62)^a^	149.17(140.10–160.12)^b^
P value		< 0.001	< 0.001	< 0.001	< 0.001

Different letters in the same column show statistical significance (^a,b^)

IgA cell count was determined to be higher in the experimental group, as was the expression density (control: 84.11 µm—experimental: 149.17 µm p < 0.001) (Fig. 4) (Table 3).

**Fig. 5:**
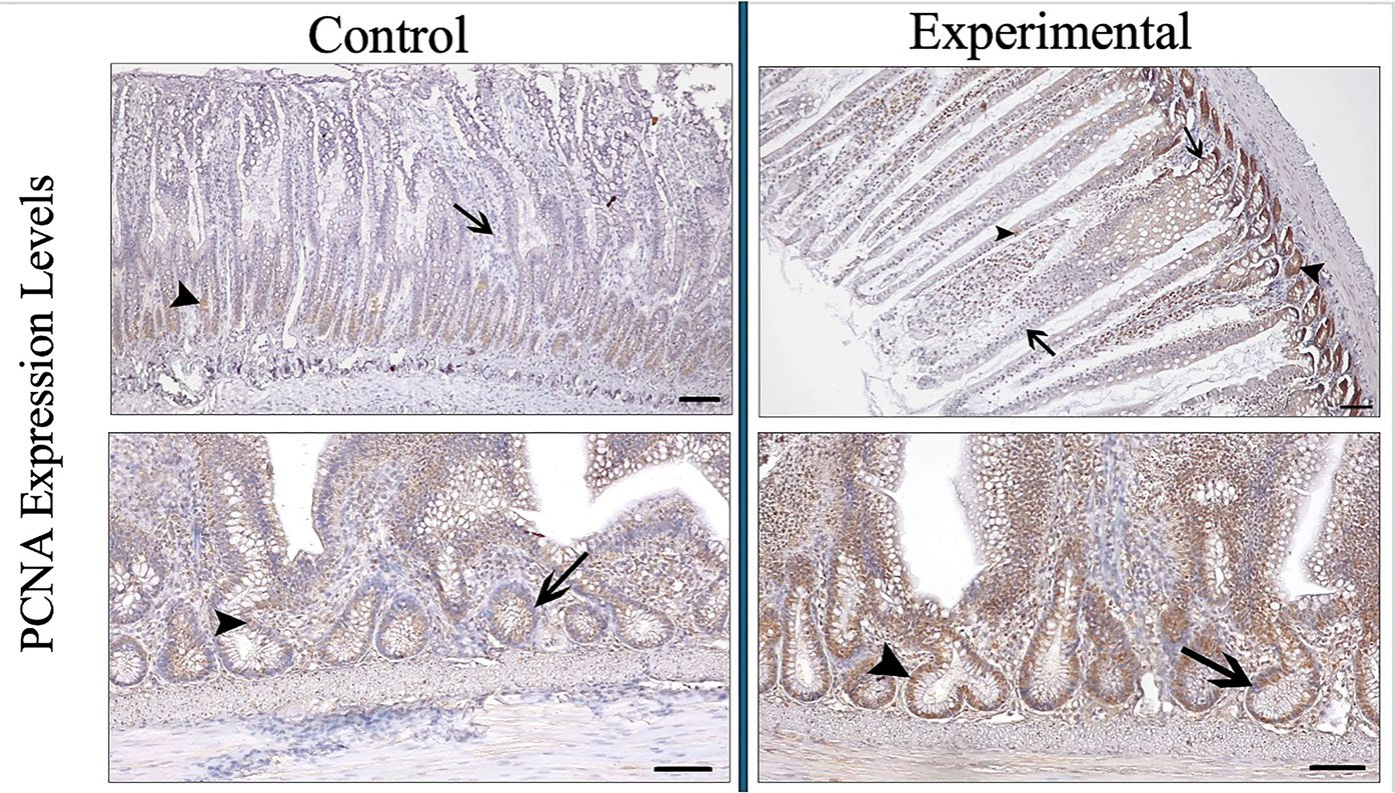
PCNA expression intensity in experimental and control groups. Arrowhead: IgA positive cells, arrow: IgA negative cells. (Bar: 50 µm)

PCNA expression intensity was determined as moderate-strong in the experimental group, while PCNA expression intensity was determined as weak in the control group (Fig. 5) (Table 3). Therefore, cell proliferation in the jejunum of aged rats treated with young plasma increased statistically (control: 1.65 µm—experimental: 2.75 µm p < 0.001).

In the PI index calculations determined in the crypt glands of the jejunum, the proliferation index in the experimental group was found to be statistically higher compared to the control group (control: 34.22 µm—experimental: 85.12 µm p < 0.001) (p < 0.001) (Fig. 5) (Table 3).

**Fig. 6:**
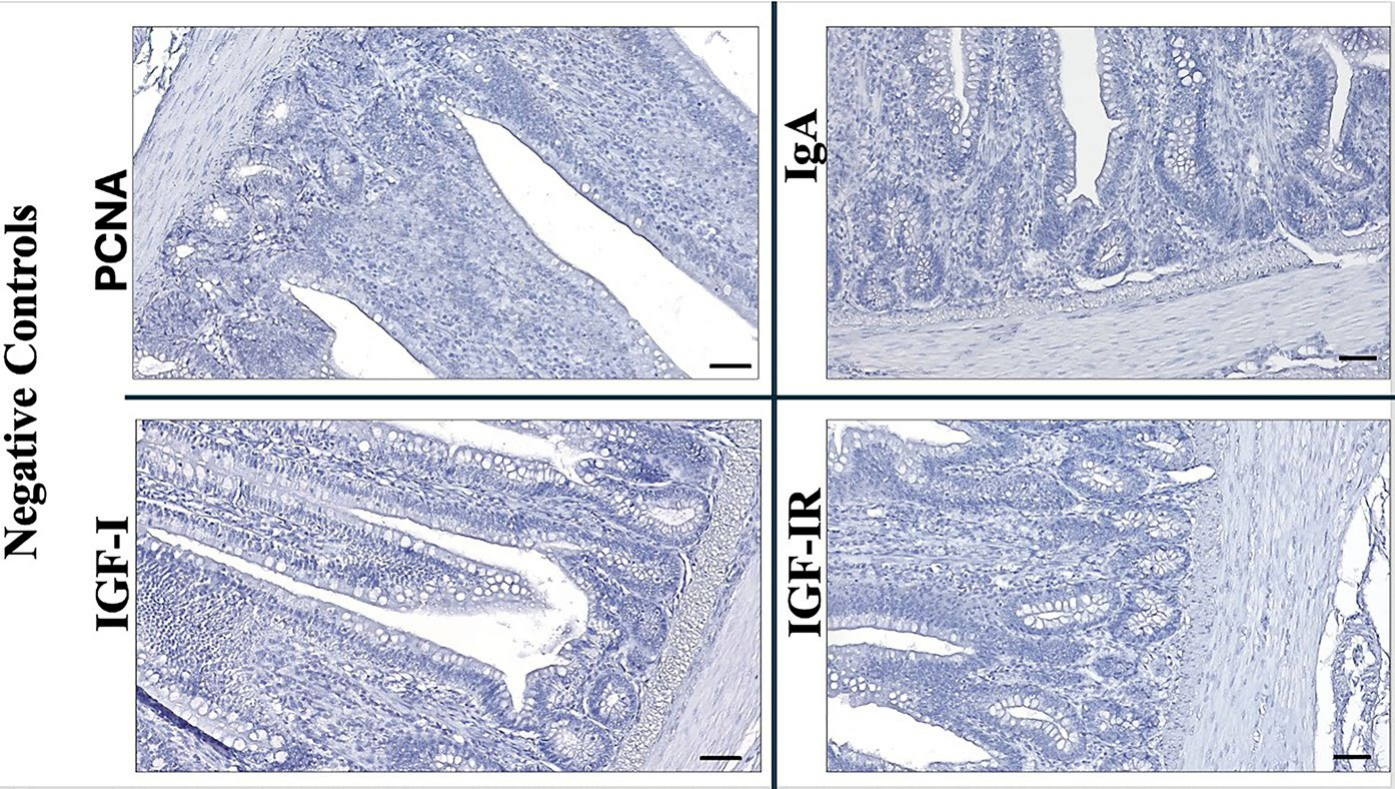
Negative control groups for PCNA, IgA, IGF-I and IGF-IR (Bar: 50 µm)

As expected, no immune reactions were observed in the negative control groups for PCNA, IGF-I, IGF-IR and IgA (Fig. 6).

## Discussion

Aging is a progressive biological process characterized by decreased cellular homeostasis, reduced regenerative capacity, and increased disease susceptibility. The gastrointestinal tract is particularly vulnerable due to constant exposure to microbial agents, dietary antigens, and inflammatory insults, resulting in epithelial deterioration, impaired nutrient absorption, and weakened immunity. Plasma therapy is earning strong recognition for its inherent capacity to improve tissue regeneration, particularly in tissue areas with a high cellular turnover like GIT which is continuously subjected to permanent exposure to microbes, and harsh luminal contents, including gastric enzymes, and acids, making it more prone to injuries. In our study, we focused on digestive system parameters by applying young plasma to aged rats. At the end of our study, we observed statistical increases in histomorphological parameters in villus height, crypt depth, total mucosal thickness, and surface absorption area in the experimental group treated with young plasma. Also, young plasma therapy induces cell proliferation and PI as we determined in the crypt cells of the jejunum, which supports digestive parameters. The increase in PI highlights the positive and rejuvenating effect of young plasma on cellular regeneration processes. Recent studies investigating the causes of many tissue damages that occur with ageing has been revealed possibility of reversibility of those effects (Ceylani et al. 2023a, 2023b; Dyall et al. 2010; Gocmez et al. 2016). These morphological changes indicate a significant improvement in the absorptive capacity of the jejunum in the treatment group. Our findings also expand on the existing body of gerontological research by demonstrating that young plasma improves the structural integrity of the gastrointestinal tract, supports cell renewal in the intestinal crypt gland, and regulates growth factor secretion and immune responses, particularly through changes in IgA levels and IGF-I and IGF-IR expression levels. Such evidence suggests potential therapeutic applications of young plasma in mitigating aging-related declines in intestinal health.

Immunoglobulin A (IgA) plays an important role in mucosal immune regulation through several mechanisms. It has active roles such as participating in immune defense by interacting with environmental antigens (e.g. bacteria, toxins, and viruses), exhibiting anti-inflammatory effects by sampling intestinal antigens to induce Th2 or regulatory T cell-biased mucosal immune responses, and maintaining homeostasis of commensals by increasing cross-talk between probiotic bacteria and intestinal mucosa (Hernández-Urbán et al. 2023). Therefore, intestinal IgA deficiency is associated with various intestinal diseases such as necrotizing enterocolitis and gastrointestinal mucositis (Corthésy 2013). Targets to increase intestinal IgA secretion are promising and aim to alleviate the pathogenesis of diseases (Ren et al. 2016). The role of IgA in controlling bacterial populations is crucial and without sufficient IgA, these populations will expand, potentially breaching the mucosal barrier and triggering both local and systemic immune responses (Fernandez et al. 2003; Suzuki et al. 2004). Ceylani et al. (2023b) demonstrated that plasma exchange between young and old rats significantly altered the gut microbiota diversity, Firmicutes/Bacteroidetes ratio, and dominant bacterial taxa, suggesting that age-related changes in plasma affect microbial homeostasis. These findings suggest that young plasma transfer shifts the gut microbiota profile of old rats toward that of young individuals, providing a more balanced microbial community, while old plasma induces dysbiosis in young recipients. This is consistent with the hypothesis that plasma-derived factors contribute to microbiota regulation, potentially through immunomodulatory mediators such as IgA. Our study suggests that the increase in IgA levels in aged rats treated with juvenile plasma is necessary to maintain intestinal integrity and enhance mucosal defense against microbial invasion.

Digestive system consists of a single cell layer of the epithelial layer, supported by the lamina propria and muscularis mucosa, forming the total tunica mucosa. In the region where nutrient absorption occurs in the epithelial tissue, the presence of villi supports absorption by increasing the surface of the epithelial layer (Helm et al. 2007). Therefore, an increase in the length of these villi structures is important for supporting absorption. Our findings demonstrated a significant increase in villus height, total mucosal thickness, crypt depth, and surface absorption area in the experimental group treated with juvenile plasma. It has been reported that the villus height is directly linked to the mitotic and proliferative activity of stem cells at the base of the crypt glands. These renewed cells migrate upwards from the crypt and renew the intestinal epithelium (Furbeyre et al. 2017). Enhanced villus height, crypt depth, total mucosal thickness after treatment with Juvenile plasma, could be linked with the presence of abundant growth factors in the plasma which are critical for cellular proliferation and tissue repair (Kaushik and Kumaran 2020). Similar to our previous study (Ceylani et al. 2023a); as a result of the administration of young plasma to the aged rats; we reported reduced inflammation and protective effects of young plasma on the intestinal tissues of aged rats and even supported cellular regeneration (Ceylani et al. 2023a). Ceylani et al. (2023a). In our study, we induced cell proliferation in the jejunum by improving the levels of microbiota-associated IgA in the jejunum with our similar application methodology. Also, previously we reported that; young plasma administration improved hepatic fibrosis, cellular degeneration, and reduced microvesicular steatosis in aged rats (Teker et al. 2023). The findings in this study also support our previous datas.

Insulin-like growth factor-I (IGF-I) is one of the important hormones that has generated great interest in gerontology. The connection between IGF-I and replacement therapy has been the centre of many studies over the last decade (Westwood et al. 2014; Sonntag et al. 2013; García-Fernández et al. 2008). IGF-I levels decrease with age and are thought to contribute to age-related declines in body activity, and as adults age, there is a decrease in IGF-I concentration (Dalcık and Dalcık 2020). IGF-I and IGF-IR, which are particularly widely distributed in the digestive tract, together with their effect on the localization of regulatory binding proteins in the intestine, make IGF-I an attractive target for regulating adaptation responses (Ohneda et al. 1997). The highest levels of IGF-IR mRNA expression are present in the fetal and early postnatal period, showing that IGF-I is primarily involved in growth (Khandwala et al. 2000). IGF-I receptor expression is significantly down-regulated in adults (Dalcık and Dalcık 2020). In the presented study, we demonstrated that young plasma treatment caused an increase in IGF-I and IGF-IR expressions and that the use of this new methodology also played a role in the regulation of hormonal mechanisms in the intestine. Demonstrating the existence of reversible effects of growth factors will also shed light on future endocrinal studies.

The study we present will contribute to the question of "How to identify and quantify the ability to tolerate, adapt, compensate and bypass age-related changes?" under the title of "Heterogeneity of the aging phenotype" presented in "Seven knowledge gaps in modern biogerontology" (Rattan 2024). The decrease in the regeneration capacity with changes in metabolism as a result of aging, the decrease in functionality of the immune system to the pathogens as an immune defense and the decrease in the expression of growth factors effective in intestinal development made us ask whether a reversible treatment approach can be applied for these effects. This shows us that in vivo preclinical laboratory experiments that can be developed on tolerating age can be effective in examining the reversible effects of age-related changes. However, the changes in metabolism as a whole and how the improvement of a single metabolic factors will have synergistic effects on other systems still needs to be investigated.

The results of this study revealed that young plasma treatment increased cell proliferation in the jejunum of aged rats with both PCNA expression intensity and PI findings. These results reveal the existence of reversible potential of tissue regeneration. In addition, this treatment method confirms the hypothesis that this treatment method can improve histomorphological parameters, especially the decline in digestion due to aging. On the other hand, our findings increased both the regressive expression of growth factor IGF-I and its receptor and the intensity and number of expressions of immune-related IgA secretions that weaken after aging. The results supporting the digestive system obtained at the end of our study provide evidence that young plasma suppresses age-related degeneration in the gastrointestinal system and may have a therapeutic effect in terms of digestive functionality in elderly; nutrient usage and absorption can be improved with this treatment method. In addition, considering that a healthy intestinal microbiota promotes a healthy life, the fact that we obtained results that support the immune system suggests that young plasma treatment may be a potential therapeutic effect on immunity. With further studies, other growth factors and immunity-related hormones and enzymes need to be examined to reach a definitive conclusion.

## Data Availability

No datasets were generated or analysed during the current study.

## References

[CR1] Allahverdi H (2024) Exploring the therapeutic potential of plasma from intermittent fasting and untreated rats on aging-induced liver damage. J Cell Mol Med 28:e18456. 10.1111/jcmm.18456 38923278 10.1111/jcmm.18456 PMC11199341

[CR2] Asmaz ED, Seyidoglu N (2022) The prevention role of Spirulina platensis (*Arthrospira platensis*) on intestinal health. Food Sci Hum Wellness 11:1342–1346. 10.1016/j.fshw.2022.04.027

[CR3] Asmaz ED, Yesilbag D, Odabasi F, Zık B (2022) Synergistic effect of feed additives on cell proliferation and morphology in quail (*Coturnix coturnix* Japonica) duodenum. J Hell Vet Med Soc 73:4575–4582. 10.12681/jhvms.27850

[CR4] Asmaz ED, Teker HT, Sertkaya ZT, Ceylani T, Genç Aİ (2025a) Effect of middle-age plasma therapy on ileum morphology, immune defense (IgA) and cell proliferation (Ki-67) of female aged rats. Histochem Cell Biol 163:17. 10.1007/s00418-024-02344-3 10.1007/s00418-024-02344-3 39688692

[CR5] Asmaz ED, Ceylani T, Genc Aİ, Sertkaya ZT, Teker HT (2025b) Plasma therapy: a novel intervention to improve age-induced decline in deudenal cell proliferation in female rat model. Biogerontology 26:57. 10.1007/s10522-025-10197-z 39920489 10.1007/s10522-025-10197-z PMC11805874

[CR6] Beharka AA, Paiva S, Leka LS, Ribaya-Mercado JD, Russell RM, Nibkin Meydani S (2001) Effect of age on the gastrointestinal-associated mucosal immune response of humans. J Gerontol A Biol Sci Med Sci 56:B218–B223. 10.1093/gerona/56.5.b218 11320102 10.1093/gerona/56.5.b218

[CR7] Cai Y, Song W, Li J et al (2022) The landscape of aging. Sci China Life Sci 65:2354–2454. 10.1007/s11427-022-2161-3 36066811 10.1007/s11427-022-2161-3 PMC9446657

[CR8] Ceylani T, Teker HT (2022) The effect of young blood plasma administration on gut microbiota in middle-aged rats. Arch Microbiol 204:541. 10.1007/s00203-022-03154-8 35930195 10.1007/s00203-022-03154-8

[CR9] Ceylani T, Teker HT, Keskin S, Samgane G, Acikgoz E, Gurbanov R (2023a) The rejuvenating influence of young plasma on aged intestine. J Cell Mol Med 27:2804–2816. 10.1111/jcmm.17926 37610839 10.1111/jcmm.17926 PMC10494294

[CR10] Ceylani T, Allahverdi H, Teker HT (2023b) Role of age-related plasma in the diversity of gut bacteria. Arch Gerontol Geriatr 111:105003. 10.1016/j.archger.2023.105003 36965198 10.1016/j.archger.2023.105003

[CR11] Conboy IM, Conboy MJ, Wagers AJ, Girma ER, Weissman IL, Rando TA (2005) Rejuvenation of aged progenitor cells by exposure to a young systemic environment. Nature 433:760–764. 10.1038/nature03260 15716955 10.1038/nature03260

[CR12] Corthésy B (2013) Role of secretory IgA in infection and maintenance of homeostasis. Autoimmun Rev 12:661–665. 10.1016/J.AUTREV.2012.10.012 23201924 10.1016/j.autrev.2012.10.012

[CR13] Crossmon G (1937) A Modification of Mallory’s connective tissue stain with a discussion of the principles involved. Anat Rec 69:33–38

[CR14] Dahly EM, Guo Z, Ney DM (2003) IGF-I augments resection-induced mucosal hyperplasia by altering enterocyte kinetics. Am J Physiol Regul Integr Comp Physiol 285:R800–R808. 10.1152/ajpregu.00014.2003 12763742 10.1152/ajpregu.00014.2003

[CR15] Dalcık H, Dalcık C (2020) Insulin-like growth factor I (IGF-I); cytoprotective effects, idea of replacement therapy in the healthy elderly subjects. MRA 8:2375–1924. 10.18103/mra.v8i6.2154

[CR16] Dyall SC, Michael GJ, Michael-Titus AT (2010) Omega-3 fatty acids reverse age-related decreases in nuclear receptors and increase neurogenesis in old rats. J Neurosci Res 88:2091–2102. 10.1002/jnr.22390 20336774 10.1002/jnr.22390

[CR17] Fernandez MI, Pedron T, Tournebize R, Olivo-Marin JC, Sansonetti PJ, Phalipon A (2003) Anti-inflammatory role for intracellular dimeric immunoglobulin a by neutralization of lipopolysaccharide in epithelial cells. Immunity 18:739–749. 10.1016/s1074-7613(03)00122-5 12818156 10.1016/s1074-7613(03)00122-5

[CR18] Firth SM, Baxter RC (2002) Cellular actions of the insulin-like growth factor binding proteins. Endocr Rev 23:824–854. 10.1210/er.2001-0033 12466191 10.1210/er.2001-0033

[CR19] Furbeyre H, van Milgen J, Mener T, Gloaguen M, Labussière E (2017) Effects of dietary supplementation with freshwater microalgae on growth performance, nutrient digestibility and gut health in weaned piglets. Animal 11:183–192. 10.1017/S1751731116001543 27452961 10.1017/S1751731116001543

[CR20] Garcia V, Catala-Gregori P, Hernandez F, Megías MD, Madrid J (2007) Effect of formic acid and plant extracts on growth, nutrient digestibility, intestine mucosa morphology, and meat yield of broilers. J Appl Poultry Res 16:555–562. 10.3382/japr.2006-00116

[CR21] García-Fernández M, Delgado G, Puche JE, González-Barón S, Castilla Cortázar I (2008) Low doses of insulin-like growth factor I improve insulin resistance, lipid metabolism, and oxidative damage in aging rats. Endocrinol 149:2433–2442. 10.1210/en.2007-1190 10.1210/en.2007-1190 18187555

[CR22] Gocmez GSS, Gacar N, Utkan T, Gacar G, Scarpace PJ, Tumer N (2016) Protective effects of resveratrol on aging-induced cognitive impairment in rats. Neurobiol Learn Mem 131:131–136. 10.1016/j.nlm.2016.03.022 27040098 10.1016/j.nlm.2016.03.022

[CR23] Guler S, Asmaz ED, Kayapunar NV, Işbilir I, Cengz ŞŞ, Yeşilbag D, Sanli AB, Gultepe EE (2022) Effects of dietary calcium, phosphorus and microbial phytase on intestinal morphology in laying hens. Turkish J Vet Anim Sci 46:293–303. 10.55730/1300-0128.4177

[CR24] Gupta P, Hu Z, Kopparapu PK, Deshmukh M, Sághy T, Mohammad M, Jin T, Engdahl C (2023) The impact of TLR2 and aging on the humoral immune response to *Staphylococcus aureus* bacteremia in mice. Sci Rep 13:8850. 10.1038/s41598-023-35970-3 37258615 10.1038/s41598-023-35970-3 PMC10232519

[CR25] Helm RM, Golden C, McMahon M, Thampi P, Badger TM, Nagarajan S (2007) Diet regulates the development of gut-associated lymphoid tissue in neonatal piglets. Neonatology 91:248–255. 10.1159/000098523 17565226 10.1159/000098523

[CR26] Hernández-Urbán AJ, Drago-Serrano ME, Cruz-Baquero A, García-Hernández AL, Arciniega-Martínez IM, Pacheco-Yépez J, Guzmán-Mejía F, Godínez-Victoria M (2023) Exercise improves intestinal IgA production by T-dependent cell pathway in adults but not in aged mice. Front Endocrinol 14:1190547. 10.3389/fendo.2023.1190547 10.3389/fendo.2023.1190547 PMC10733478 38130396

[CR27] Kaushik A, Kumaran MS (2020) Platelet-rich plasma: the journey so far ! Indian Dermatol Online J 11:685–692. 10.4103/idoj.IDOJ_369_19 33235832 10.4103/idoj.IDOJ_369_19 PMC7678541

[CR28] Khandwala HM, Mc Cutcheon IE, Flyvbjerg A, Friend KE (2000) The effects of Insulin-like growth factors on tumorigenesis and neoplastic growth. Endocr Rev 21:215–244. 10.1210/edrv.21.3.0399 10857553 10.1210/edrv.21.3.0399

[CR29] Kuemmerle JF (2012) Insulin-like growth factors in the gastrointestinal tract and liver. Endocrinol Metab Clin North Am 41:409–423. 10.1016/j.ecl.2012.04.018 22682638 10.1016/j.ecl.2012.04.018 PMC3372868

[CR30] Loffredo FS, Steinhauser ML, Jay SM, Gannon J, Pancoast JR, Yalamanchi P, Sinha M, Dall’Osso C, Khong D, Shadrach JL, Miller CM, Singer BS, Stewart A, Psychogios N, Gerszten RE, Hartigan AJ, Kim MJ, Serwold T, Wagers AJ, Lee RT (2013) Growth differentiation factor 11 is a circulating factor that reverses age-related cardiac hypertrophy. Cell 153:828–839. 10.1016/j.cell.2013.04.015 23663781 10.1016/j.cell.2013.04.015 PMC3677132

[CR31] Mantell MP, Ziegler TR, Adamson WT, Roth JA, Zhang W, Frankel W, Bain A, Chow JC, Smith RJ, Rombeau JL (1995) Resection-induced colonic adaptation is augmented by IGF-I and associated with upregulation of colonic IGF-I mRNA. Am J Physiol 269:G974–G980. 10.1152/ajpgi.1995.269.6.G974 8572229 10.1152/ajpgi.1995.269.6.G974

[CR32] Merchant HA, Rabbie SC, Varum FJ, Afonso-Pereira F, Basit AW (2014) Influence of ageing on the gastrointestinal environment of the rat and its implications for drug delivery. Eur J Pharm Sci 62:76–85. 10.1016/j.ejps.2014.05.004 24834990 10.1016/j.ejps.2014.05.004

[CR33] Ohneda K, Ulshen MH, Fuller CR (1997) Enhanced growth of small bowel in transgenic mice expressing human insulin-like growth factor I. Gastroenterol 112:444–454. 10.1053/gast.1997.v112.pm9024298 10.1053/gast.1997.v112.pm9024298 9024298

[CR34] Pollak MN, Schernhammer ES, Hankinson SE (2004) Insulin-like growth factors and neoplasia. Nat Rev Cancer 4:505–518. 10.1038/nrc1387 15229476 10.1038/nrc1387

[CR35] Rattan SIS (2024) Seven knowledge gaps in modern biogerontology. Biogerontology 25:1–8. 10.1007/s10522-023-10089-0 38206540 10.1007/s10522-023-10089-0

[CR36] Ren W, Wang K, Yin J, Chen S, Liu G, Tan B, Wu G, Bazer FW, Peng Y, Yin Y (2016) Glutamine-induced secretion of intestinal secretory immunoglobulin A: a mechanistic perspective. Front Immunol 7:503. 10.3389/FIMMU.2016.00503 27933057 10.3389/fimmu.2016.00503 PMC5121228

[CR37] Ruttanavut J, Yamauchi K (2010) Growth performance and histological alterations of intestinal villi in broilers fed dietary mixed minerals. Asian J Anim Sci 4:96–106. 10.3923/ajas.2010.96.106

[CR38] Sonntag WE, Deak F, Ashpole N, Toth P, Csiszar A, Freeman W, Ungvari Z (2013) Insulin-like growth factor-1 in CNS and cerebrovascular aging. Front Aging Neurosci 5:27. 10.3389/fnagi.2013.00027 23847531 10.3389/fnagi.2013.00027 PMC3698444

[CR39] Sun Y, Li Q, Kirkland JL (2022) Targeting senescent cells for a healthier longevity: the roadmap for an era of global aging. Life Med 1:103–119. 10.1093/lifemedi/lnac030 36699942 10.1093/lifemedi/lnac030 PMC9869767

[CR40] Suzuki K, Meek B, Doi Y, Muramatsu M, Chiba T, Honjo T, Fagarasan S (2004) Aberrant expansion of segmented filamentous bacteria in IgA-deficient gut. Proc Natl Acad Sci USA 17(101):1981–1986. 10.1073/pnas.0307317101 10.1073/pnas.0307317101 PMC357038 14766966

[CR41] Teker HT, Ceylani T, Keskin S, Samgane G, Mansuroglu S, Baba B, Allahverdi H, Acıkgoz E, Gurbanov R (2023) Age-related differences in response to plasma exchange in male rat liver tissues: insights from histopathological and machine-learning assisted spectrochemical analyses. Biogerontology 24:563–580. 10.1007/s10522-023-10032-3 37017896 10.1007/s10522-023-10032-3

[CR42] Tripathi SS, Kumar R, Arya JK, Rizvi SI (2021) Plasma from young rats injected into old rats induce antiaging effects. Rejuvenation Res 24(3):206–212. 10.1089/rej.2020.2354 33161876 10.1089/rej.2020.2354

[CR43] Villeda SA, Luo J, Mosher KI, Zou B, Britschgi M, Bieri G, Stan TM, Fainberg N, Ding Z, Eggel A, Lucin KM, Czirr E, Park JS, Couillard-Després S, Aigner L, Li G, Peskind ER, Kaye JA, Quinn JF, Galasko DR, Xie XS, Rando TA, Wyss-Coray T (2011) The ageing systemic milieu negatively regulates neurogenesis and cognitive function. Nature 31(477):90–94. 10.1038/nature10357 10.1038/nature10357 PMC3170097 21886162

[CR44] Wang K, Liu H, Hu Q, Wang L, Liu J, Zheng Z, Zhang W, Ren J, Zhu F, Liu G-H (2022) Epigenetic regulation of aging: implications for interventions of aging and diseases. Sig Transduct Target Ther 7:3–74. 10.1038/s41392-022-01211-8 10.1038/s41392-022-01211-8 PMC9637765 36336680

[CR45] Westwood AJ, Beiser A, Decarli C, Harris TB, Chen TC, He XM, Roubenoff R, Pikula A, Au R, Braverman LE, Wolf PA, Vasan RS, Seshadri S (2014) Insulin-like growth factor-1 and risk of Alzheimer dementia and brain atrophy. Neurology 82:1613–1619. 10.1212/WNL.0000000000000382 24706014 10.1212/WNL.0000000000000382 PMC4013812

[CR46] Yang SB, Qin YJ, Ma X, Luan WM, Sun P, Ju AQ, Duan AY, Zhang YN, Zhao DH (2021) Effects of in ovo injection of astragalus polysaccharide on the intestinal development and mucosal immunity in broiler chickens. Front Vet Sci 30:738816. 10.3389/fvets.2021.738816 10.3389/fvets.2021.738816 PMC8435677 34527718

[CR47] Yesilbag D, Abdullahoglu E, Urkmez E, Acar A, Asmaz D, Kara M (2022) Evaluation of the effects of different natural dietary feed additives on performance and intestinal histomorphology in quails. J Hell Vet Med Soc 73:4407–4416. 10.12681/jhvms.27265

[CR48] Zık B, Kurnaz H, Güler S, Asmaz ED (2019) Effect of tamoxifen on the Notch signaling pathway in ovarian follicles of mice. Biotech Histochem 94:410–419. 10.1080/10520295.2019.1580387 31305178 10.1080/10520295.2019.1580387

